# Severe perioperative lactic acidosis in a pediatric patient with glycogen storage disease type Ia: a case report

**DOI:** 10.1186/s40981-023-00683-z

**Published:** 2023-12-20

**Authors:** Tamayo Takahashi, Kana Oue, Eiji Imado, Mitsuru Doi, Yoshitaka Shimizu, Mitsuhiro Yoshida

**Affiliations:** 1 Department of Dental Anesthesiology, Division of Oral and Maxillofacial Surgery and Oral Medicine, Hiroshima University Hospital, 1-2-3 Kasumi, Minami-Ku, Hiroshima, 734-8551 Japan; 2 Department of Dental Anesthesiology, Graduate School of Biomedical and Health Sciences, Hiroshima University, 1-2-3 Kasumi, Minami-Ku, Hiroshima, 734-8551 Japan

**Keywords:** Glycogen storage disease, Carbohydrate metabolism, Lactic acidosis, Hypoglycemia, Hepatomegaly

## Abstract

**Background:**

Glycogen storage disease (GSD) is a group of rare inherited metabolic disorders caused by enzyme deficiencies in glycogen catabolism. GSD type Ia is a congenital deficiency of the enzyme responsible for the final step in glucose production by glycolysis, resulting in impaired carbohydrate metabolism.

**Case presentation:**

A 14-year-old boy with GSD type Ia was scheduled for a maxillary cystectomy under general anesthesia. He was taking oral sugars such as uncooked cornstarch regularly to prevent hypoglycemia. Perioperatively, glucose was administered via the peripheral vein for fasting; however, severe lactic acidosis occurred. He also developed hypercapnia because of intraoperative poor ventilation caused by hepatomegaly.

**Conclusions:**

We experienced a child with GSD type Ia who developed severe lactic acidosis despite continuous glucose infusion. Further studies are required to determine appropriate perioperative management for patients with GSD, including fasting glucose administration.

## Background

In glycogen storage disease (GSD), glycogen accumulates in the liver and muscles caused by a congenital deficiency of enzymes involved in the synthesis or degradation of glycogen [1]. GSD type I is an extremely rare inherited disorder of carbohydrate metabolism that occurs at a birth rate of approximately 1/100,000–1/400,000 [2], and it is divided into types Ia and Ib. GSD type Ia (GSD Ia) accounts for 80% of GSD type I cases and is caused by a biallelic mutation in the glucose-6-phosphatase catalytic-subunit-encoding gene (*G6PC*) and is considered the most severe type of GSD [3]. Glucose-6-phosphatase (G6Pase) is related to the final step in the production of glucose from glycogenesis. The loss of G6Pase function disrupts glucose metabolism homeostasis, and patients with GSD Ia are at risk for severe hypoglycemia throughout their lifetime [3–5]. G6Pase deficiency can also cause glycogen accumulation in the liver and other organs, hyperuricemia, lactic acidosis [6], growth retardation due to frequent hypoglycemia, and epistaxis due to decreased platelet function [3]. Perioperative anesthetic management increases the risk of metabolic and homeostatic imbalances, particularly hypoglycemia and metabolic acidosis. However, only a few studies have reported the anesthetic management of patients with this disease because of its rarity [6–12]. Herein, we report the anesthetic management of a pediatric patient with GSD Ia who exhibited severe lactic acidosis during surgery under general anesthesia.

## Case presentation

**Fig. 1:**
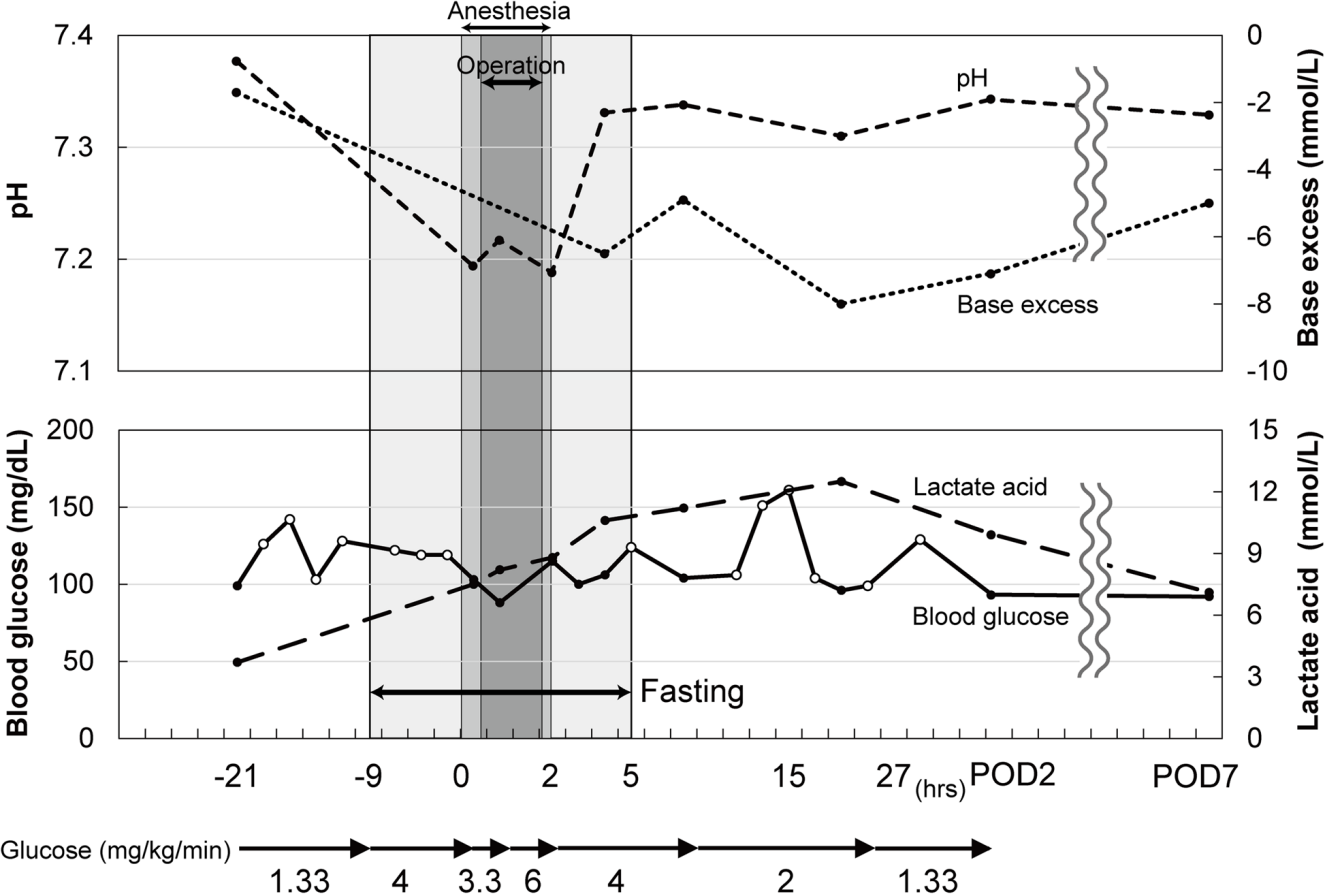
Perioperative blood gas analysis and dosage of glucose administration. Each value was measured by blood gas analysis and by a simple blood glucose meter. Black circles indicate values measured by blood gas analysis and white circles indicate values measured by a simple blood glucose meter. Glucose administration was started preoperatively via the peripheral vein. Severe lactic acidosis was observed from the beginning of surgery and continued until the second postoperative day. POD: postoperative day

A 14-year-old boy (height, 124 cm; weight, 25.2 kg) with GSD Ia was scheduled for a maxillary cystectomy under general anesthesia. He exhibited no abnormal physical and mental development except short stature; however, but had frequent epistaxis. To prevent hypoglycemia, he had been taking, in addition to his three regular meals, frequently a formula for GSD treatment (low-fat milk with lactose and fructose removed) and uncooked cornstarch, except during sleeping. Preoperative chest radiography showed an elevated diaphragm because of hepatomegaly. Laboratory data were remarkable for aspartate aminotransferase of 94 IU/L, alanine aminotransferase of 98 IU/L, γ-glutamyl transpeptidase of 154 IU/L, triglyceride of 1203 mg/dL, and total cholesterol of 272 mg/dL.Infusion of 5% glucose solution was started 21 h before surgery through his peripheral vein, with 1.3 mg/kg/min of glucose, which was increased to 4 mg/kg/min before 9 h of surgery when preoperative fasting was initiated. Blood glucose level before entering the operating room was 119 mg/dL (Fig. 1).

After entering the operating room, an infusion of Ringer’s acetate solution containing 5% glucose, corresponding to glucose 3.3 mg/kg/min, was initiated. Anesthesia was induced with remifentanil, thiamylal, sevoflurane, and rocuronium, followed by orotracheal intubation, and maintained with sevoflurane and remifentanil. Blood gas analysis immediately after anesthesia induction revealed severe lactic acidosis with pH of 7.194 and lactate of 7.5 mmol/L, which was not corrected by infusion of sodium bicarbonate or an increase of glucose infusion rate to 6 mg/kg/min. Intraoperative ventilation was set in the pressure-controlled ventilation mode with a tidal volume of 170–300 mL, peak inspiratory pressure of 15–17 cmH_2_O, positive end-expiratory pressure of 5 cmH_2_O, and rate of 12–16/min in the head-down position. Arterial carbon dioxide tension was 45–52 mmHg. On completion of surgery, the tracheal tube was removed after full recovery from anesthesia, and the patient was transferred to the ward.

Once in the ward, glucose was continued at 4 mg/kg/min. Two hours after surgery, in addition to glucose administration, he resumed intake of uncooked cornstarch and the special formula. Thereafter, the glucose dose was gradually decreased. Immediate postoperative blood gas analysis showed improvement in acidosis. Lactate levels rose to 12.5 mmol/L on the morning of the first postoperative day but then gradually decreased. No hypoglycemia was observed in the postoperative period.

## Discussion

Currently, no fundamental treatment has been determined for GSD type I, and diet is the mainstay of treatment. The main goal is to maintain blood glucose levels and prevent metabolic abnormalities, which requires diet therapy (small frequent meals every 3–4 h, lactose and sucrose elimination, and fructose restriction), special formula for GSD treatment (low-fat milk with lactose and fructose removed) and uncooked cornstarch, and frequent or continuous nighttime supplementation [13–16]. In patients with GSD Ia, glycogenolysis and glycogenesis are accelerated during hypoglycemia; however, glucose is not synthesized from glucose-6-phosphate (G6P), which flows into the glycolytic system and is converted to lactate, resulting in hyperlactatemia [5]. Perioperatively, hypoglycemia due to restricted oral intake, large fluctuations in blood glucose levels with surgical invasiveness, and enhancement of glycogenolysis and lactic acid production by catecholamines are induced. Although close perioperative monitoring of blood glucose and lactate levels before elective surgery is important for the management of GSD type I [13], appropriate glucose dosage to maintain perioperative blood glucose and lactate levels in patients with GSD is still unknown.

Several studies reported perioperative glucose doses for patients with GSD type Ia (Table 1). Despite continuous glucose infusion in all of those patients, four patients developed lactic acidosis and hypoglycemia perioperatively [9, 11], and five developed lactic acidosis without hypoglycemia [7, 10, 11].

**Table 1:** Case mix and surgical and anesthetic characteristics of patients with glycogen storage disease type Ia

Reference	Age, years (sex)	Surgery	Anesthetic	Type of infusion and glucose dosage	Others
Cox JM (1968) [7]	17	Resection of chondromas of the femoral head	STP, S, Hal, N_2_O	B) G5 solution	Lactic acidosis
Bevan JC. (1980) [8]	9 (M)	Femoral osteotomy	STP, S, N_2_O, TC, Hal	A) G: 6.7 mg/kg/min B) G17 solution C) G17 solution	N/A
Bustamante SE, et al. (2006) [6]	4 (F)	Tonsillectomy	Sev, F, Ro, Prop, RF, N_2_O	A) G5 solution and 0.45NS	Postoperative acute pancreatitis
Oshita A, et al. (2008) [9]	19 (M), 31 (F)	Liver segmentectomy	None reported	B) G: 3.3 mg/kg/min C) G: 6.6–9.8 mg/kg/min or G: 6.6 mg/kg/min + rapid-acting insulin 5 IU/h	Hypoglycemia, metabolic acidosis
Takeuchi M, et al. (2010) [10]	7 (F)	STA-MCA Bypass surgery	MZ, F, Ro, N_2_O, Sev	B) G5-7 solution	Lactic acidosis, decreased ventilation volume
Mollet-Boudjemline A, et al. (2011) [11]	34 (M) 23 (F) 24 (F) 26 (F) 20 (M) 18 (M)	Hepatectomy	None reported	A) G: 4 mg/kg/min + corticoid B) G: 6.0 mg/kg/min C) G: decreased by 1 mg/kg/min every 8 h	Lactic acidosis in 5 of 6 patients, hypoglycemia in 2 of them
Mikuriya Y, et al. (2012) [12]	39 (M)	Liver segmentectomy	None reported	B) G: 3.3 mg/kg/min C) G: 6.6–9.8 mg/kg/min or G: 6.6 mg/kg/min + rapid-acting insulin 5 IU/h	N/A

A) Fasting before surgery, B) during surgery, and C) after surgery

*G* glucose, *Gx* x% glucose, i.e., G6.7, 6.7% glucose, *NS* normal saline, *LR* lactate Ringer’s solution

*F* fentanyl, *Hal* halothane, *MZ* midazolam, *N*_*2*_*O* nitrous oxide, *prop* propofol, *RF* remifentanil, *Ro* rocuronium, *S* suxamethonium, *Sev* sevoflurane, *STA-MCA* superficial temporal artery to middle cerebral artery, *STP* sodium thiopental, *TC* tubocurarine

In this case, glucose infusion had been started the day before surgery and continued during and after surgery. Despite normal blood glucose levels, severe acidosis occurred, which might be induced by the following mechanism. Accumulated G6P due to G6Pase deficiency is metabolized to pyruvate via glycolysis. Although pyruvate is normally consumed by entering into the citric acid cycle in mitochondria, mitochondrial dysfunction in patients with GSD Ia may lead to lactic acid production from pyruvic acid [17]. Additionally, perioperative stress-induced hormone secretion may lead to increased G6P production from glycogen. Another possible cause of perioperative severe acidosis is insufficient perioperative glucose dosing relative to the requirements of the perioperative organism. According to previous reports, glucose doses were 3.3–6.7, 6.0–9.8, and 6.6 mg/kg/min before, during, and after surgery [9]. Although lactic acidosis might have been prevented by a larger dose of perioperative glucose in our case, it should be administered from a central venous line and whether an invasive procedure for inserting it is required in such a minimally invasive oral surgery is unclear.

Diaphragmatic elevation by the head-down position in addition to preexisting hepatomegaly would have limited tidal volume [10], leading to hypercapnia and worsening of acidosis in this case. Consequently, the partial pressure of carbon dioxide in the blood increased, which may have contributed to the worsening of the acidosis.

Bleeding tendency is a problem in patients with GSD type I because of impaired platelet function [4, 13]. Bleeding complications were reported in 23% of patients with GSD I, and perioperative bleeding tendency should be noted [13]. In this case, oral intubation was performed instead of nasal intubation because of frequent epistaxis. No abnormal postoperative bleeding was observed.

Patients with GSD Ia often present with hypertriglyceridemia, and pancreatitis is an important complication [18, 19]. Acute pancreatitis is reported after general anesthesia with propofol in a patient with GSD Ia [6]. Because acute pancreatitis may be caused by propofol in patients with hypertriglyceridemia [20], its use should be avoided in such patients. We used thiamylal instead of propofol with sevoflurane, resulting in no postoperative complications including pancreatitis.

A few studies have reported the perioperative management of GSD because of its rarity and low prevalence; thus, more studies are needed to determine the appropriate perioperative management for patients with GSD, including glucose infusion during fasting. Standard perioperative management must be established to manage complications and improve the care of patients with GSD undergoing surgery or receiving intensive care.

## Data Availability

Data sharing is not applicable to this article as no datasets were generated or analyzed during the current study.

## Abbreviations

GSD: Glycogen storage disease
G6P: Glucose-6-phosphate
G6Pase: Glucose-6-phosphatase
G6PC: Glucose-6-phosphatase catalytic-subunit-encoding gene
